# A new group of glycoside hydrolase family 13 α-amylases with an aberrant catalytic triad

**DOI:** 10.1038/srep44230

**Published:** 2017-03-13

**Authors:** Fean D. Sarian, Štefan Janeček, Tjaard Pijning, Ihsanawati, Zeily Nurachman, Ocky K. Radjasa, Lubbert Dijkhuizen, Dessy Natalia, Marc J. E. C. van der Maarel

**Affiliations:** 1 Biochemistry Research Division, Faculty of Mathematics and Natural Sciences, Institut Teknologi Bandung, Jl. Ganesha 10, Bandung, 40132, Indonesia; 2 Aquatic Biotechnology and Bioproduct Engineering, Engineering and Technology institute Groningen (ENTEG), University of Groningen, Nijenborgh 4, 9747 AG Groningen, The Netherlands; 3 Laboratory of Protein Evolution, Institute of Molecular Biology, Slovak Academy of Sciences, Dubravska cesta 21, SK-84551 Bratislava, Slovakia; 4 Department of Biology, Faculty of Natural Sciences, University of SS. Cyril and Mehtodius, Nam. J. Herdu 2, SK-91701 Trnava, Slovakia; 5 X-Ray Crystallography, Groningen Biomolecular Sciences and Biotechnology Institute (GBB), University of Groningen, Nijenborgh 7, 9747 AG Groningen, The Netherlands; 6 Department of Marine Science, Faculty of Fishery and Marine Science, Diponegoro University, Semarang 50275, Central Java, Indonesia; 7 Microbial Physiology, Groningen Biomolecular Sciences and Biotechnology Institute (GBB), University of Groningen, Nijenborgh 7, 9747 AG Groningen, The Netherlands

## Abstract

α-Amylases are glycoside hydrolase enzymes that act on the α(1→4) glycosidic linkages in glycogen, starch, and related α-glucans, and are ubiquitously present in Nature. Most α-amylases have been classified in glycoside hydrolase family 13 with a typical (β/α)_8_-barrel containing two aspartic acid and one glutamic acid residue that play an essential role in catalysis. An atypical α-amylase (BmaN1) with only two of the three invariant catalytic residues present was isolated from *Bacillus megaterium* strain NL3, a bacterial isolate from a sea anemone of Kakaban landlocked marine lake, Derawan Island, Indonesia. In BmaN1 the third residue, the aspartic acid that acts as the transition state stabilizer, was replaced by a histidine. Three-dimensional structure modeling of the BmaN1 amino acid sequence confirmed the aberrant catalytic triad. Glucose and maltose were found as products of the action of the novel α-amylase on soluble starch, demonstrating that it is active in spite of the peculiar catalytic triad. This novel BmaN1 α-amylase is part of a group of α-amylases that all have this atypical catalytic triad, consisting of aspartic acid, glutamic acid and histidine. Phylogenetic analysis showed that this group of α-amylases comprises a new subfamily of the glycoside hydrolase family 13.

α-Amylases are ubiquitously present in nature. They act on the α(1→4) glycosidic linkages in glycogen, starch, and related α-glucans and thereby play an important role in the digestion of starch in humans, plants and microorganisms^1^,^2^,^3^. Most α-amylases belong to glycoside hydrolase (GH) family 13^4^, constituting 30 different reaction and product specificities including, glycoside hydrolases (EC 3.2.1.x), glucanotransferases (EC 2.4.1.x and EC 2.4.99.16), and isomerases (EC 5.4.99.x), all sharing a conserved structural scaffold^4^,^5^. The crystal structure of Taka α-amylase A from *Aspergillus oryzae* (TAA), the first experimentally determined three-dimensional (3D) structure of α–amylase^6^, revealed that α-amylases have three characteristic domains: A, B, and C^6^,^7^. The A domain containing the catalytic residues is the most conserved domain, with a typical (β/α)_8_- or TIM-barrel comprised of eight stranded parallel β-sheet surrounded by eight α-helices. Domain B is inserted between the third β-strand and the third α-helix of the (β/α)_8_-barrel and varies in length and structure. The C domain folds into eight antiparallel β-strands, is connected to the A domain by loops and seems to be an independent domain with unknown function^5^,^8^. Despite low similarity between the amino acid sequences of α-amylases from animals, plants, and microorganisms, the GH13 enzymes share seven highly conserved regions^7^ that are involved in the formation of the catalytic site. The α-amylase active site is located in an open cavity between the A and B domains, and contains the invariably carboxylic acid Asp206, Glu230 and Asp297 (TAA numbering) being essential for catalysis, acting as the nucleophile, and as the general acid/base and transition state stabilizer, respectively^6^.

Twenty years ago, several α-amylases and related enzymes composed of a (β/α)_7_-barrel (an irregular TIM-barrel domain) were classified into the family GH57; more recently also the family GH119 was established^9^,^10^. Both of these enzyme families are at present considerably smaller than GH13 and only few members have been characterized in detail^11^. The first determined 3D structure of GH57 was that of the 4-α-glucanotransferase from *Thermococcus litoralis* (TLGT). X-ray crystallography supported by site-directed mutagenesis of TLGT revealed that it has two catalytic residues, Glu123 and Asp214, as the catalytic nucleophile and the general acid/base, respectively^12^. No 3D structure is currently available for GH119 members. In addition to the structural differences between GH13 and GH57-GH119 family members, there are also distinctive conserved regions between these families^9^. The GH57 and GH119 families share five conserved sequence regions^13^.

Several microbial strains isolated from a unique land-locked marine lake located in Kakaban island, part of the Derawan Islands, East Kalimantan, Indonesia, were screened for the production of α-amylases. The lake originally was the lagoon of an atoll, formed by corals over a period of two million years. As a result of movements in the earth’s crust the coral reef was raised above the sea level, trapping 5 km^2^ of seawater within a 50 meter high ridge, effectively creating a land-locked marine lake^14^. One of the isolates, *Bacillus megaterium* NL3, contained an active GH13 α-amylase with only the general acid/base residue (Glu231) at the conserved position. Amino acid sequence alignments and 3D homology modeling showed that the nucleophile may be shifted one position downstream (Asp203) and that the transition state stabilizing residue is not the canonical Asp but a His residue (His294). Phylogenetic analysis clustered this new α-amylase and its homologs, which also possess the incomplete GH13 catalytic machinery, as a separate branch in family GH13, representing a novel subfamily.

## Results and Discussion

### Screening of Kakaban lake isolates producing extracellular amylases

Eight of twenty bacterial isolates from Kakaban landlocked marine lake tested positive for the hydrolysis of starch by producing a clear halo around their colonies on red-dyed amylopectin agar plates. Isolate NL3 showed the largest clearing zone, indicating a relatively high α-amylase activity and was selected for further study. 16S rDNA sequence analysis showed that strain NL3 was most closely related to *Bacillus megaterium*. This result was in agreement with biochemical and physiological properties (data not shown) and hence the selected isolate was designated as *B. megaterium* NL3. The culture medium of strain NL3 showed activity towards soluble starch. The 50–80% ammonium sulphate precipitate of the culture medium gave a single protein band with molecular mass of approximately 55 kDa on SDS-PAGE in combination with activity staining with soluble starch (data not shown).

### Molecular identification of the NL3 amylase

Using degenerate α-amylase specific primers and inverse PCR, a DNA fragment of 2.3 kb was obtained from genomic DNA of strain NL3. Analysis of the nucleotide sequence of this fragment showed that an open reading frame of 1515 bp with clear α-amylase sequence similarity was present. This gene was designated as *bmaN1*. A putative ribosomal binding site (RBS) corresponding to the AGGAGG sequence located 12 nucleotides upstream of the start codon was predicted. A probable catabolite responsive element (CRE) was found together with possible −10 (TATAAT) and −35 (TTAACA) regions. The CRE sequence showed only one mismatch in the last position when compared to the consensus sequence (TGT/AAANCGNTNA/TCA)^15^. The BmaN1 polypeptide deduced is 505 amino acid residues in length with a clear putative signal peptide sequence of 23 residues preceding the mature enzyme, as predicted by SignalP 4.0 Server^16^. The molecular weight and *p*I of BmaN1 were predicted using ExPASy server (http://web.expasy.org/protparam) as 56934 Da and 9.05, respectively. The full-length DNA sequence of the putative α-amylase gene of *B. megaterium* NL3, *bmaN1*, has been deposited in the GenBank database^17^ under the accession no. AGT45938.

### *In silico* analysis of BmaN1 and its homologues

**Figure 1:**
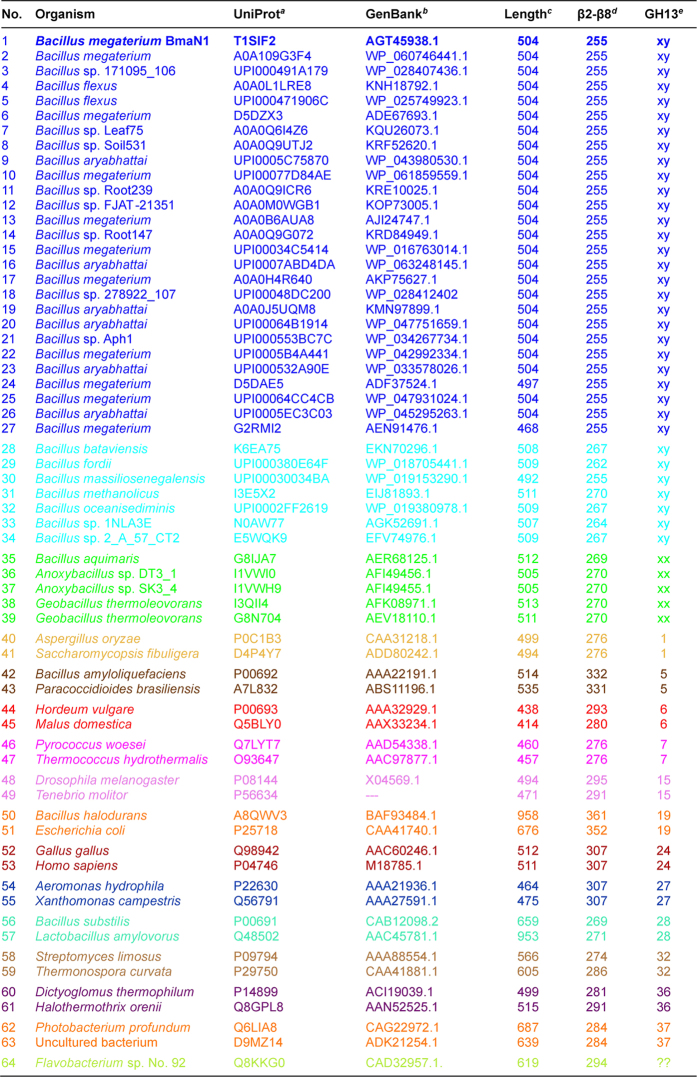
α-Amylases used in the present study^*a*^. ^*a*^The list involves: (i) the members of the newly proposed GH13 subfamily xy represented by the “α-amylase” from *Bacillus megaterium* BmaN1 (Nos 1–27) and its closely related homologues (Nos 28–34) - probably intermediates between BmaN1 and the α-amylase from *Bacillus aquimaris* BaqA - caught by BLAST; (ii) the members of the recently proposed GH13 subfamily xx^19^ represented by the BaqA (Nos 35–39); (iii) representatives of the individual GH13 subfamilies with the specificity of α-amylases - subfamilies GH13_1, 5, 6, 7, 15, 19, 24, 27, 28, 32, 36 and 37 (Nos 40–63); and (iv) the currently unassigned cyclomaltodextrinase (GH13_??; No. 64); for details, see the Materials and methods section. ^*b,c*^Accession numbers from the UniProt (UniParc) and GenBank sequence databases, respectively. ^*d*^The length of the entire amino acid sequence of the protein. ^*e*^The length of the polypeptide chain spanning the segment from the beginning of the CSR-VI (strand β2) to the end of the CSR-VII (strand β8). ^*f*^The GH13 subfamily (if available).

**Figure 2:**
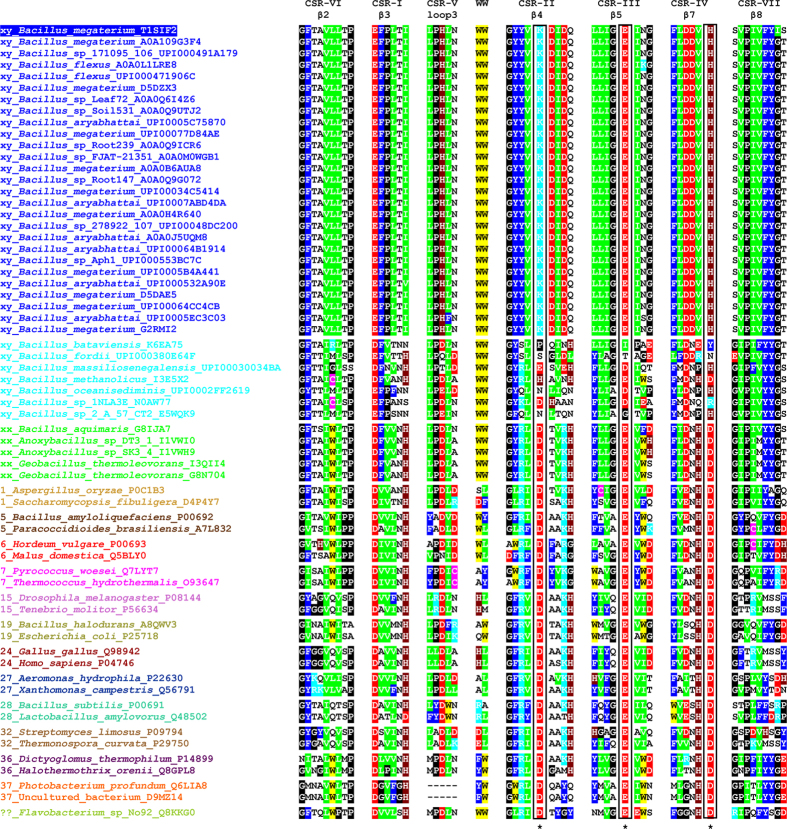
Sequence alignment of CSRs of studied family GH13 enzymes with focus on the novel α-amylase subfamily. The two consecutive tryptophans characteristic for the novel α-amylase subfamily are also shown. Colour code for the selected residues: W, yellow; F, Y - blue; V, L, I - green; D, E - red; R, K - cyan; H - brown; C - magenta; G, P - black. The positions of the three catalytic residues are boxed and signified by asterisks under the alignment. The label of the protein source consists of the name of the organisms and the UniProt (UniParc) accession number. The number at the beginning of the protein source label indicates the number of known (already established) GH13 subfamily. For the newly proposed BmaN1 GH13 subfamily, the label “xy” is used; similarly (“xx”) for until now non-defined subfamily around the BaqA. The alignment of all 64 enzymes spanning the sequence segment from the beginning of the strand β2 (CSR-VI) to the end of the strand β8 (CSR-VII) is shown in Fig. S2.

The BLAST search using the BmaN1 amino acid sequence as a query resulted in retrieving of more than 30 highly similar sequences of putative α-amylases (Fig. 1) some of which have already been classified in the family GH13^18^. Although all of them possess variations in the three residues forming the family GH13 catalytic machinery, it is possible to divide them into two groups: (i) the first, larger group (Nos 1–27 in Fig. 1) with Lys202 and His294 in the positions of the catalytic nucleophile and transition state stabilizer, respectively (instead of normally occurring aspartates); and (ii) a second, smaller group (Nos 28–34 in Fig. 1) exhibiting substitutions in positions of the entire catalytic triad, but rather without an obvious regularity (Fig. 2). While the sequences of the members of the former group are almost identical to BmaN1, those of the latter one are slightly different (Fig. 2). Interestingly, there is a strictly conserved aspartic acid residue succeeding the “strange” lysine, which corresponds with the position of the catalytic nucleophile (Fig. 2). The sequences of both these groups, proposed here to constitute a novel GH13 subfamily xy around the α-amylase from *B. megaterium* BmaN1, are all highly similar to those of α-amylases around the α-amylase from *B. aquimaris* BaqA (Nos 35–39 in Fig. 1) suggested recently to define also a new and independent GH13 subfamily xx^19^. The main difference between the α-amylases around the BmaN1 and those around BaqA is that the BaqA α-amylase and the members of its subfamily possess the complete catalytic machinery (Fig. 2) characteristic for the α-amylase family GH13^7^. The other feature of interest is the presence of a tryptophan pair in both BmaN1 and BaqA (Fig. 2) between the CSR-V (loop 3) and CSR-II (strand β4), located in the helix α3 of the catalytic (β/α)_8_-barrel^19^.

In addition to the incomplete catalytic machinery mentioned above, the most striking differences of BmaN1 and its close homologues discriminating them from other well-established GH13 subfamilies with the α-amylase specificity (Fig. 2) are the presence of a glutamic acid instead of aspartate at the beginning of the CSR-I (strand β3) and in addition the position of the histidine in the middle of the CSR-V (loop3), a position usually occupied by aspartic acid^20^. With regard to alignment of the representative α-amylases studied here (Fig. 1), its substantial part covering almost the entire (β/α)_8_-barrel including domain B clearly document a very close homology of both eventual GH13 subfamilies, i.e. BmaN1 and BaqA. All these sequences (Nos 1–39 in Fig. 1) go well together exhibiting their own pattern of the alignment in comparison to remaining α-amylases that represent well-established GH13 subfamilies (Nos 40–63 in Fig. 1). Of note is also the fact that the small group of putative α-amylases with irregular substitutions in catalytic positions (Nos 28–34 in Fig. 1) exhibits obviously a higher similarity to the BaqA subfamily than to that around BmaN1, especially in domain B (preceding the CSR-V in loop3) as well as in the segment preceding the CSR-IV at strand β7 (Fig. 2). The cyclomaltodextrinase from *Flavobacterium* sp. No. 92^21^ was added to the comparison as an interesting example since it was recently found to possess the pair of adjacent tryptophan residues^19^, typical for both BmaN1 and BaqA (Fig. 2). This is of interest because the cyclomaltodextrinase belongs to GH13 members intermediate between subfamilies of oligo-1,6-glucosidases and neopullulanases^22^ that are closely related to α-amylases from the subfamily GH13_36 that, however, do not possess the tryptophan pair (Fig. 2).

**Figure 3:**
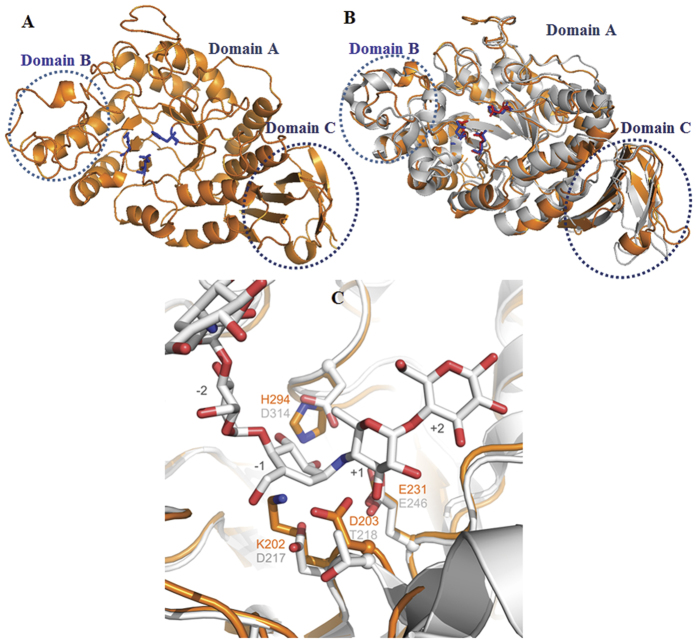
Structural comparison of BmaN1 of *B. megaterium* NL3 and GTA of *Geobacillus thermoleovorans*. (**A**) BmaN1 model structure, (**B**) Structure of BmaN1 (orange) superimposed on GTA (grey) structure, (**C**) Active-site region in a superposition of BmaN1 with GTA including the acarbose bound in its subsites −2 to +2 (white carbon atoms). Active-center residues of BmaN1 (orange) and GTA (grey) are given as stick models and labeled in orange (BmaN1) and black (GTA).

A topological alignment of BmaN1 and the putative α-amylases of *B. megaterium* DSM319, *Bacillus* sp. 278922, *B. flexus, B. aryabhattai, B. megaterium* WSH-002, and GTA was made (Fig. S1). Almost all β-strands and α-helices of the TIM barrel in domain A and the Greek key motif in domain C are conserved in these α-amylases. A model of the 3D structure of BmaN1 was generated by the PHYRE server^23^ and visualized by the MacPymol software^24^ (Fig. 3). The BmaN1 protein displayed 40% homology (100% confidence, 85% sequence coverage) with the X-ray crystal structure of *Geobacillus thermoleovorans* α-amylase (GTA, PDB code: 4E2O)^25^ which was used as a template for the modeling. The comparison between the model and the GTA crystal structure revealed that the global topology is almost the same (Fig. 3). The BmaN1 protein model folds into three distinct domains: a central A domain of 366 residues harboring a (β/α)_8_ barrel, with an irregular loop domain of 37 residues (domain B) connecting the third β-sheets strand and the third α-helix of the barrel. The C domain of 79 residues has an eight-stranded anti-parallel β-sandwich-like fold (Fig. 3). The (β/α)_8_ barrel is quite similar to the (β/α)_8_-barrel found in maltogenic amylase from *Pseudomonas saccharophila* and *Bacillus stearothermophilus*^26^, in that there is an additional helix between Aα6 and Aβ7, which is a three-turn helix lying nearly parallel with the Aα6 strand.

Superposition of acarbose-bound GTA with the BmaN1 model demonstrated that of the three catalytic residues found in GH13 α-amylases, only residue Glu231 of BmaN1 superimposes with the corresponding residue in GTA (Glu246), and presumably is the general acid/base in BmaN1 (Fig. 3). As already concluded from sequence alignments, two of the three catalytic residues are not conserved in BmaN1. Lys202 replaces the catalytic aspartate (Asp217 of GTA); however, Asp203 directly downstream of the lysine is positioned nearby and has its carboxylic acid side chain pointing into the presumed substrate-binding groove (Fig. 3). Furthermore, at the position corresponding to the nucleophile, His294 replaces the transition-state stabilizing aspartate residue (Asp314) found in α-amylases. The absence of any one of the catalytic residues normally causes partial or complete loss of hydrolysis activity^27^. Remarkably, the mutant α-amylase from *Xanthomonas campestris* truncated from the C-terminal part of domain B and thus lacking any of the three conserved catalytic residues, still exhibited starch-hydrolyzing activity^28^, but that observation has never been supported by other examples.

### Phylogeny of BmaN1 and other α-amylases

**Figure 4:**
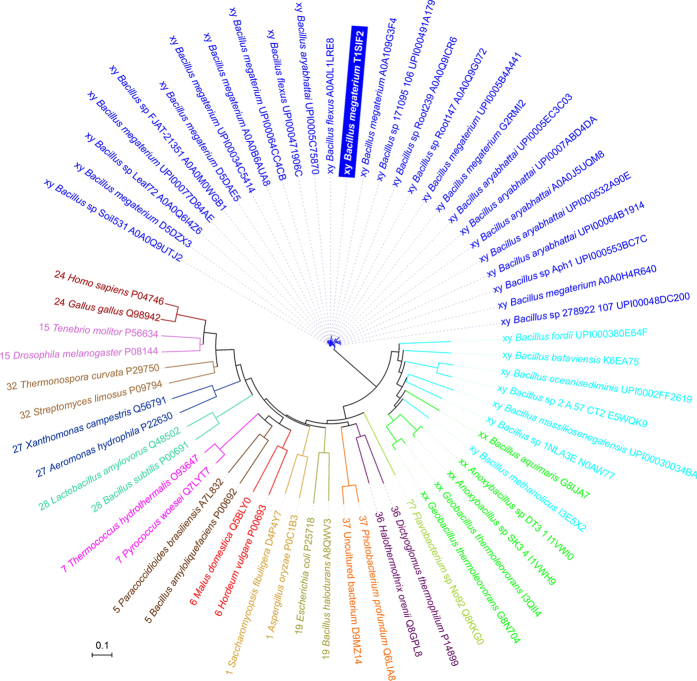
Evolutionary tree of studied family GH13 enzymes with focus on the novel α-amylase subfamily around the BmaN1. The label of the protein source consists of the name of the organisms and the UniProt (UniParc) accession number preceded by GH13 subfamily indication. The tree is based on the alignment shown in Fig. S2.

The evolutionary relatedness of the α-amylase from *B. megaterium* BmaN1, representing all its homologues with lysine and histidine in positions of the catalytic nucleophile and transition state stabilizer, respectively (Fig. 2; Nos 1–27 in Fig. 1), to members of the recently proposed GH13 subfamily around the BaqA (Nos 35–39 in Fig. 1) as well as to representatives of remaining well-established GH13 subfamilies with α-amylase specificity (Nos 40–63 in Fig. 1), is shown in the evolutionary tree (Fig. 4). It is clear that both subfamilies BmaN1 and BaqA are most closely related to each other among all family GH13 α-amylases. Furthermore, a small group of putative α-amylases with irregular substitutions in catalytic positions (Nos 28–34 in Fig. 1) may be considered as an intermediary connection between both BmaN1 and BaqA subfamilies since, despite the lack of complete family GH13 catalytic machinery (similar to BmaN1), they cluster together with representatives of the BaqA subfamily (Fig. 4). One of the most convincing sequence-structural features characteristic for all these α-amylases is the presence of the pair of adjacent tryptophan residues in helix α3 of the catalytic (β/α)_8_-barrel (Fig. S2)^19^. Interestingly, the *Flavobacterium* sp. No. 92 cyclomaltodextrinase (with the tryptophan pair) is positioned in the evolutionary tree between the subfamilies of BmaN1 and BaqA and all other remaining GH13 families with the α-amylase specificity (Fig. 4).

### *BmaN1* encodes an active exo-acting α-amylase

**Figure 5:**
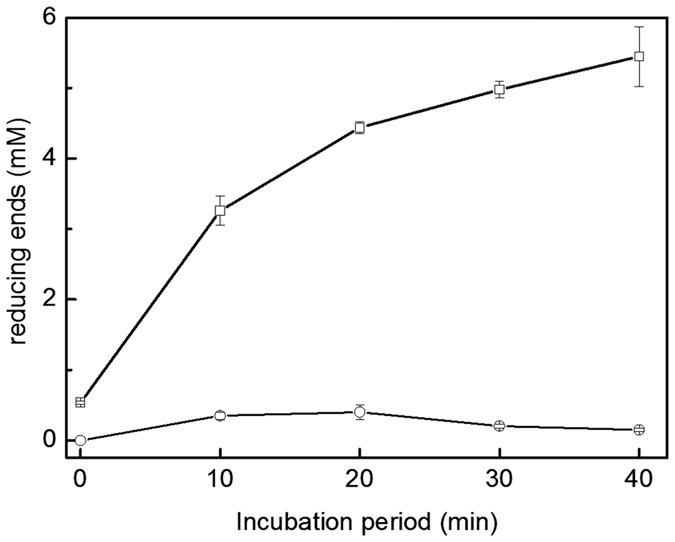
Incubation of BmaN1 (open squares) with soluble starch; open circles: control (empty vector). 1% (w/v) soluble starch and 12.5 μg/mL of the BmaN1 protein were incubated for various time at 55 °C. Each data point represents the means of triplicate experiments.

**Figure 6:**
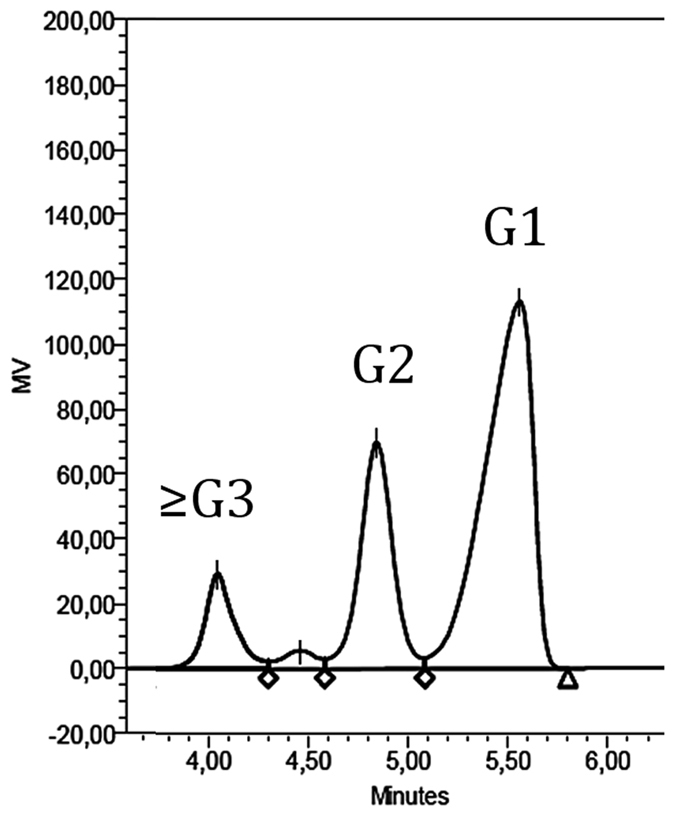
HPLC analysis for hydrolyzed products of soluble starch by BmaN1. A reaction mixture containing 1% (w/v) soluble starch, 12.5 μg/mL of BmaN1, 50 mM maleate buffer pH 6.0 in a total volume of 0.1 mL was incubated at 55 °C. G1, glucose; G2, maltose; ≥G3, maltooligosaccharides.

The gene encoding BmaN1 was cloned in vector pMM1525 and this recombinant plasmid was transformed to *B. megaterium* MS941. A transformant with clear α-amylase activity, as detected on starch plates by iodine staining, was selected and grown in liquid medium. The culture medium was saturated with 50–80% concentrations of ammonium sulphate to purify the BmaN1 α-amylase enzyme. The molecular weight of the partially purified BmaN1 was estimated to be 55 kDa as judged from activity staining after protein renaturing on SDS-PAGE gels (Fig. S3). In contrast, no band was observed in the culture supernatant of *B. megaterium* MS941 carrying pMM1525 without any insert (Fig. S3). Amylolytic activity of BmaN1 was measured spectrophotometrically by incubating it with soluble starch and measuring the increase in the amount of reducing sugars released over a period of 40 min (Fig. 5). A clear increase in reducing ends was observed, resulting in an activity of 8.4 U/mg of protein. BmaN1 was found to be most active at 55 °C and pH 6.0. The main end products formed from soluble starch were glucose and maltose, indicating an exo-acting mode of action. Minor amounts of longer chain maltooligosaccharides were also found (Fig. 6). This mode of action is very similar to that of the amylase from *Bacillus* sp. IMD 435 that releases glucose and maltose as the major products on hydrolysis of both soluble starch and raw corn starch^29^.

The results presented above indicate that the substitution of an aspartate residue by a histidine, a positively charged amino acid, still gives (some) amylase activity. The reaction mechanism of BmaN1 may be essentially different from the general catalytic reaction mechanism of α-amylases. Further experiments are needed to demonstrate whether the His residue indeed is one of the catalytic residues of α-amylases.

## Methods

### Materials

All chemicals used were reagent grade and were obtained from either Fermentas (Maryland, USA) or Difco Laboratories (New Jersey, USA).

### Bacterial strains, plasmids, and growth conditions

Twenty microbial strains (gift of Prof. Ocky Karna Radjasa of Diponegoro University, Indonesia) that had been isolated from Kakaban landlocked marine lake (Derawan Islands, East Kalimantan, Indonesia) were screened for extracellular α-amylase activity. The isolates were cultured in marine broth (MB) medium containing 0.25% (w/v) yeast extract, and 0.5% (w/v) peptone in filtered sea water (Seaworld, Ancol, Jakarta, Indonesia) at 30 °C. *B. megaterium* MS941 (MoBiTec, Germany) and *Escherichia coli* TOP10 were grown at 37 °C in LB medium (1% (w/v) Bacto-tryptone, 1% (w/v) NaCl and 0.5% (w/v) yeast extract). Ampicillin and tetracyclin were used at concentrations of 100 μg/ml and 12 μg/ml, respectively. The medium was autoclaved at 120 °C for 30 min prior to adding the antibiotics. Plasmid pGEM-T (Promega, USA) was used for PCR product cloning, whereas pMM1525 (MoBiTec, Germany) was used as expression vector.

### Screening of α-amylase producing bacteria

Bacterial isolates were inoculated on MB agar plates supplemented with 1.0% (v/v) red-dyed amylopectin^30^ and then incubated at 30 °C for 24 h. The appearance of a clear zone against a red background was indicative for the production of α-amylase activity. The positive isolates were then subjected to a second screening round using MB agar plates containing 1.0% (w/v) potato or wheat starch. A clearing zone around the bacterial colony indicated that the starch was hydrolyzed and thus that the isolate produced extracellular amylase activity.

### Bacterial identification

The isolate showing the largest clearing zone on starch-agar plate was identified by 16SrDNA sequencing. Chromosomal DNA was isolated using Wizard Genomic DNA Purification (Promega). The 16S rDNA gene was amplified by PCR using universal primers UniB1 and BactF1 (Supplementary Table 1). The resulted 1.4 kb fragment was sequenced using the dideoxy-chain termination method (Macrogen, South Korea). The bacterial isolate was identified by aligning the 16s rDNA sequences with other known bacteria using NCBI BLASTn (http://www.ncbi.nlm.nih.gov). 16S rDNA gene sequence was submitted to GenBank.

### Cloning of the α-amylase-encoding gene and plasmid construction

Two degenerate primers (Table S1) were designed based on the amino acid sequences of the well-conserved regions (region VI-VII) of α-amylases from several Bacilli. The first α-amylase gene fragment was amplified by polymerase chain reactions (PCR), using chromosomal DNA from *B. megaterium* NL3 as a template and the two degenerate primers. The PCR products were inserted into pGEM-T vector (Promega) and transformed into *E. coli* TOP10. Plasmid DNA of the transformed *E. coli* TOP10 was isolated and the nucleotide sequence of the inserted DNA was determined using the dideoxy-chain termination method (Macrogen). The resulting nucleotide sequence was used to design a set of primers, NL3_SP8-invF1 and NL3_SP8-invR1 (Table S1), to amplify parts of α-amylase gene beyond the conserved region. The chromosomal DNA was partially digested with *EcoR*V and then self-ligated using T4 DNA ligase (Fermentas). Inverse PCR (iPCR) was performed with Dream *Taq* polymerase (Fermentas) and the primers listed in Supplementary Table 1 using the self-ligated DNA fragment as a template. Analysis of sequence data and sequence similarity searches was performed using the BLAST program of the National Center for Biotechnology Information (NCBI). Primers pMM-NL3-F and pET/MM-NL3-R (Table S1) were used to amplify the complete open reading frame of the α-amylase gene which was designated as *bmaN1*.

### Transformation of *B. megaterium*

The recombinant plasmid containing the α-amylase gene, pMM1525-bmaN1, was transformed into the expression host, *B. megaterium* MS941. The transformation procedure was essentially conducted as described by Puyet *et al*. with some modifications^31^. A 0.5 ml protoplast suspension was added to a tube containing 5.0 μg DNA and 1.5 ml PEG-P (40% (w/v) PEG6000 in 1x SMMP) for each transformation and incubated for 2 min at room temperature. SMMP medium contains 3.5% (w/v) AB3 (Antibiotic medium no. 3, Difco), 1 M sucrose, 40 mM disodium maleic acid and 40 mM MgCl_2_ (pH adjusted to 6.5 before autoclaving for 12 min) and prepared freshly before use. To the mixture, 5.0 ml SMMP was added and mixed by rolling the tube carefully. Cells were harvested by centrifugation at 2,700 ×  *g* for 10 min at room temperature and the supernatant was poured off immediately. The pellets were resuspended with 0.5 ml SMMP and incubated at 37 °C for 90 min with gentle shaking or rolling of tubes (max. 100 rpm). Then, 50 to 200 μl of cells were added into top agar and mixed gently by rolling the tube. The mixture was poured on a pre-warmed plate of LB containing 12 μg/ml of tetracycline and incubated at 37 °C overnight.

### Expression and partial purification of recombinant α-amylase

α-Amylase was produced by growing the *B. megaterium* harboring recombinant plasmids in 20 ml of LB medium supplemented with 12 μg/ml tetracycline at 37 °C with shaking. The overnight culture was transferred into fresh media and incubated until the 546-nm absorbance reached 0.8–1.0. Subsequently, expression was induced by adding 1% (v/v) xylose, and the culture was incubated at 18 °C with constant shaking at 150 rpm for 24 h. Cells were removed by centrifugation (6000 × *g*, 10 min) and the resulted supernatant was subjected to ammonium sulphate precipitation at a saturation value up to 80%. The precipitate was dissolved and dialyzed against 50 mM maleate buffer pH 6.0 at 4 °C. This partially purified α-amylase was used for further studies.

### Gel electrophoresis and activity staining

SDS-PAGE was carried out as described by Laemmli^32^ and gels were then stained with Coomassie Brilliant Blue (Bio-Rad). For α-amylase activity test, the protein samples were separated by SDS-PAGE containing 1% soluble starch. After electrophoresis, SDS was removed by washing the gel with water followed by 10 min incubation at room temperature. This was repeated twice. The gels were then immersed in the enzyme reaction buffer (50 mM maleate buffer pH 6.0) for 4.0 h at 55 °C and then stained with KI/I_2_ solution for 10 min and followed by rinsing with water. The α-amylase activity was detected as a clear zone against a purple background.

### Enzyme assay

The amylase activity assay was conducted using the 3,5-dinitrosalicylic acid (DNS) method described by Miller (1955) with a slight modification^33^. Briefly, the assay was performed by adding 25 μl of enzyme sample into 25 μl of 1% (w/v) soluble starch (Fermentas, USA) in 50 mM of the appropriate buffer and then incubated at 55 °C for 10 min. To the reaction mixture, 50 μl of DNS reagent was added. The absorbance at 500 nm was measured and then the amount of reducing sugar-end was calculated using a glucose standard curve. One unit of α-amylase activity was defined as the amount of enzyme that releases 1 μmol of reducing sugar per min under the assay conditions. The protein concentration was determined using the Lowry method and bovine serum albumin as the standard.

### Analysis of sugars

The starch hydrolysis products were analyzed by high-performance liquid chromatography (HPLC). Aliquots of 100 μl of enzyme solution were incubated at 55 °C in the presence of 1% (w/v) soluble starch, maleate buffer 50 mM. After specific time intervals, samples were withdrawn and hydrolysis was stopped by incubation at 90 °C for 10 min. After centrifugation at 12000 × *g* for 10 min at 4 °C, the products were then analyzed by HPLC (Aminex^®^ HPX-87H system). The separated hydrolysis products were identified by calculating based on peak areas compared to standard glucose, maltose, and purified maltooligosaccharide (Sigma).

### Bioinformatics

The sequences eventually forming the new GH13 subfamily xy were collected based on protein BLAST^34^ searches against the non-redundant database using the entire amino acid sequence of *Bacillus megaterium* NL3 α-amylase BmaN1 (UniProt accession No.: T1SIF2) as well as on previous bioinformatics analyses when the BLAST was performed with the *Bacillus aquimaris* α-amylase BaqA^19^,^35^. The main criterion applied for the selection was the lack of at least one residue from the catalytic triad characteristic for the α-amylase family GH13. In addition to BmaN1 and its closely related homologues, five experimentally characterized α-amylases from the recently proposed GH13 subfamily around the *B. aquimaris* α-amylase BaqA^19^,^35^ were added. These α-amylases – BaqA from *B. aquimaris*^35^, ASKA and ADTA from *Anoxybacillus* sp.^36^,^37^, GTA and GTA-II from *Geobacillus thermoleovorans*^25^,^38^,^39^ – exhibit a high degree of sequence similarity with BmaN1, but possess the complete GH13 catalytic machinery^19^. The entire set was finally completed by two selected representatives from well-established GH13 subfamilies with the α-amylase specificity, i.e. 1, 5, 6, 7, 15, 19, 24, 27, 28, 32, 36 and 37^19^ including also the related but until now unclassified cyclomaltodextrinase from *Flavobacterium* sp. No. 192^21^ so that the final number of studied enzymes and hypothetical proteins was 64 (Fig. 1).

All 64 GH13 sequences were retrieved from GenBank^17^ and UniProt^40^ sequence databases and the set was aligned using the program Clustal-Omega^41^ available at the European Bioinformatics Institute’s web-site (http://www.ebi.ac.uk/). A subtle manual tuning was done in order to maximize similarities, especially with regard to aligning the individual CSRs. The boundaries of the CSRs were defined based on previous bioinformatics studies^19^,^20^. The evolutionary tree was constructed based on the final alignment of the sequence segment corresponding to a 255-residue long region of BmaN1 α-amylase spanning almost the entire catalytic (β/α)_8_-barrel domain including the domain B from the beginning of the CSR-VI (strand β2; starting with Gly79) to the end of the CSR-VII (strand β8; ending with Ser333). The tree was calculated as a Phylip-tree type using the neighbour-joining clustering and the bootstrapping procedure - the number of bootstrap trials used was 1,000^42^ implemented in the Clustal-X package^43^, and then displayed with the program iTOL^44^.

The 3D structure of BmaN1 was predicted by QuickPhyre structure program server (http://www.sbg.bio.ic.ac.uk/phyre)^23^. Structural modeling of the BmaN1 was performed based on the crystal structures of α-amylase of *G. thermoleovorans* [PDB access code: 4E20]. The generated BmaN1 structures were displayed and drawn by MacPymol.

## Additional Information

**How to cite this article**: Sarian, F. D. *et al*. A new group of glycoside hydrolase family 13 α-amylases with an aberrant catalytic triad. *Sci. Rep.* **7**, 44230; doi: 10.1038/srep44230 (2017).

**Publisher's note:** Springer Nature remains neutral with regard to jurisdictional claims in published maps and institutional affiliations.

## Supplementary Material

Supplementary Dataset 1

## References

[b1] Van der Maarel M. J. E. C., van der Veen B., Uitdehaag J. C. M., Leemhuis H. & Dijkhuizen L. Properties and applications of starch-converting enzymes of the α-amylase family. J. Biotechnol. 94, 137–155 (2002). 11796168 10.1016/s0168-1656(01)00407-2

[b2] Butterworth P. J., Warren F. J. & Ellis P. R. Human α-amylase and starch digestion: An interesting marriage. Starch - Stärke 63, 395–405 (2011).

[b3] Zeeman S. C., Kossmann J. & Smith A. M. Starch: its metabolism, evolution and biotechnological modification in plants. Ann. Rev. Plant Biol. 61, 209–234 (2010). 20192737 10.1146/annurev-arplant-042809-112301

[b4] Cantarel B. L. . The Carbohydrate-Active EnZymes database (CAZy): an expert resource for Glycogenomics. Nucleic Acids Res. 37, D233–238 (2009). 18838391 10.1093/nar/gkn663 PMC2686590

[b5] MacGregor E. A., Janeček Š. & Svensson B. Relationship of sequence and structure to specificity in the α-amylase family of enzymes. Biochim. Biophys. Acta - Protein Struct. Mol. Enzymol. 1546, 1–20 (2001). 10.1016/s0167-4838(00)00302-2 11257505

[b6] Matsuura Y., Kusunoki M., Harada W. & Kakudo M. Structure and possible catalytic residues of Taka-amylase A. J. Biochem. 95, 697–702 (1984). 6609921 10.1093/oxfordjournals.jbchem.a134659

[b7] Janeček Š., Svensson B. & MacGregor E. A. α-Amylase: an enzyme specificity found in various families of glycoside hydrolases. Cell. Mol. Life Sci. 71, 1149–1170 (2014). 23807207 10.1007/s00018-013-1388-z PMC11114072

[b8] MacGregor E. A. α-Amylase structure and activity. J. Protein Chem. 7, 399–415 (1988). 3267138 10.1007/BF01024888

[b9] Janeček Š. & Kuchtová A. *In silico* identification of catalytic residues and domain fold of the family GH119 sharing the catalytic machinery with the α-amylase family GH57. FEBS Lett. 586, 3360–3366 (2012). 22819817 10.1016/j.febslet.2012.07.020

[b10] Blesák K. & Janeček Š. Two potentially novel amylolytic enzyme specificities in the prokaryotic glycoside hydrolase α-amylase family GH57. Microbiology 159, 2584–2593 (2013). 24109595 10.1099/mic.0.071084-0

[b11] Henrissat B. & Davies G. Structural and sequence-based classification of glycoside hydrolases. Curr. Opin. Struct. Biol. 7, 637–644 (1997). 9345621 10.1016/s0959-440x(97)80072-3

[b12] Imamura H. . Crystal structures of 4-α-glucanotransferase from *Thermococcus litoralis* and its complex with an inhibitor. J. Biol. Chem. 278, 19378–19386 (2003). 12618437 10.1074/jbc.M213134200

[b13] Zona R., Chang-Pi-Hin F., O’Donohue M. J. & Janeček Š. Bioinformatics of the glycoside hydrolase family 57 and identification of catalytic residues in amylopullulanase from *Thermococcus hydrothermalis*. Eur. J. Biochem. 271, 2863–2872 (2004). 15233783 10.1111/j.1432-1033.2004.04144.x

[b14] Radjasa O. K., Limantara L. & Sabdono A. Antibacterial activity of a pigment producing-bacterium associated with *Halimeda sp.* from island-locked marine lake kakaban, Indonesia. J. Coast. Dev. 12, 100–104 (2019).

[b15] Hueck C. J. & Hillen W. Catabolite repression in *Bacillus subtilis*: a global regulatory mechanism for the Gram-positive bacteria? Mol. Microbiol. 15, 395–401 (1995). 7540244 10.1111/j.1365-2958.1995.tb02252.x

[b16] Petersen T. N., Brunak S., von Heijne G. & Nielsen H. SignalP 4.0: discriminating signal peptides from transmembrane regions. Nat Meth 8, 785–786 (2011). 10.1038/nmeth.1701 21959131

[b17] Benson D. A. . GenBank. Nucleic Acids Res. 43, D30–5 (2015). 25414350 10.1093/nar/gku1216 PMC4383990

[b18] Lombard V. . The carbohydrate-active enzymes database (CAZy) in 2013. Nucleic Acids Res. 42, D490–D495 (2013). 24270786 10.1093/nar/gkt1178 PMC3965031

[b19] Janeček Š., Kuchtová A. & Petrovičová S. A novel GH13 subfamily of α-amylases with a pair of tryptophans in the helix α3 of the catalytic TIM-barrel, the LPDlx signature in the conserved sequence region V and a conserved aromatic motif at the C-terminus. Biologia 70, 1284–1294 (2015).

[b20] Janeček Š. How many conserved sequence regions are there in the α-amylase family? Biologia 57 (Suppl. 11), 29–41 (2002).

[b21] Fritzsche H. B., Schwede T. & Schulz G. E. Covalent and three-dimensional structure of the cyclodextrinase from *Flavobacterium sp.* no. 92. Eur. J. Biochem. 270, 2332–2341 (2003). 12752453 10.1046/j.1432-1033.2003.03603.x

[b22] Majzlová K., Pukajová Z. & Janeček Š. Tracing the evolution of the α-amylase subfamily GH13_36 covering the amylolytic enzymes intermediate between oligo-1,6-glucosidases and neopullulanases. Carbohydr. Res. 367, 48–57 (2013). 23313816 10.1016/j.carres.2012.11.022

[b23] Kelley L. A. . The Phyre2 web portal for protein modeling, prediction and analysis. Nat. Protoc. 10, 845–858 (2015). 25950237 10.1038/nprot.2015.053 PMC5298202

[b24] Schrodinger LLC. The PyMOL molecular graphics system, Version 1.3r1 (2010).

[b25] Mok S.-C. . Crystal structure of a compact α-amylase from *Geobacillus thermoleovorans*. Enzyme Microb. Technol. 53, 46–54 (2013). 23683704 10.1016/j.enzmictec.2013.03.009

[b26] Jespersen H. M., MacGregor E. A., Sierks M. R. & Svensson B. Comparison of the domain-level organization of starch hydrolases and related enzymes. Biochem. J. 280, 51–55 (1991). 1741756 10.1042/bj2800051 PMC1130598

[b27] Nielsen J. E. & Borchert T. V. Protein engineering of bacterial α-amylases. Biochim. Biophys. Acta - Protein Struct. Mol. Enzymol. 1543, 253–274 (2000). 10.1016/s0167-4838(00)00240-5 11150610

[b28] Ke T. . A mutant α-amylase with only part of the catalytic domain and its structural implication. Biotechnol. Lett. 29, 117–122 (2007). 17091385 10.1007/s10529-006-9208-2

[b29] Hamilton L. M., Kelly C. T. & Fogarty W. M. Production and properties of the raw starch-digesting α-amylase of *Bacillus sp.* IMD 435. Process Biochem. 35, 27–31 (1999).

[b30] Jørgensen S., Vorgias C. E. & Antranikian G. Cloning, sequencing, characterization, and expression of an extracellular α-amylase from the hyperthermophilic archaeon *Pyrococcus furiosus* in *Escherichia coli* and *Bacillus subtilis*. J. Biol. Chem. 272, 16335–16342 (1997). 9195939 10.1074/jbc.272.26.16335

[b31] Puyet A. . A simple medium for rapid regeneration of *Bacillus subtilis* protoplasts transformed with plasmid DNA. FEMS Microbiol. Lett. 40, 1–5 (1987).

[b32] Laemmli U. K. Cleavage of structural proteins during the assembly of the head of bacteriophage T4. Nature 227, 680–685 (1970). 5432063 10.1038/227680a0

[b33] Miller G. L. Use of dinitrosalicylic acid reagent for determination of reducing sugar. Anal. Chem. 31, 426–428 (1959).

[b34] Altschul S. F. . Basic local alignment search tool. J. Mol. Biol. 215, 403–10 (1990). 2231712 10.1016/S0022-2836(05)80360-2

[b35] Puspasari F. . Raw starch-degrading α-amylase from *Bacillus aquimaris* MKSC 6.2: isolation and expression of the gene, bioinformatics and biochemical characterization of the recombinant enzyme. J. Appl. Microbiol. 114, 108–120 (2013). 23020612 10.1111/jam.12025

[b36] Chai Y. Y., Rahman R. N., Illias R. M. & Goh K. M. Cloning and characterization of two new thermostable and alkalitolerant α-amylases from the *Anoxybacillus* species that produce high levels of maltose. J. Ind. Microbiol. Biotechnol. 39, 731–741 (2012). 22246222 10.1007/s10295-011-1074-9

[b37] Chai K. P. . Crystal structure of *Anoxybacillus* α-amylase provides insights into maltose binding of a new glycosyl hydrolase subclass. Sci. Rep. 6, 23126 (2016). 26975884 10.1038/srep23126 PMC4791539

[b38] Finore I. . Purification, biochemical characterization and gene sequencing of a thermostable raw starch digesting α-amylase from *Geobacillus thermoleovorans* subsp. stromboliensis subsp. nov. World J. Microbiol. Biotechnol. 27, 2425–2433 (2011).

[b39] Mehta D. & Satyanarayana T. Domain C of thermostable α-amylase of *Geobacillus thermoleovorans* mediates raw starch adsorption. Appl. Microbiol. Biotechnol. 98, 4503–4519 (2014). 24413972 10.1007/s00253-013-5459-8

[b40] UniProt Consortium. UniProt: a hub for protein information. Nucleic Acids Res. 43, D204–12 (2015). 25348405 10.1093/nar/gku989 PMC4384041

[b41] Sievers F. . Fast, scalable generation of high-quality protein multiple sequence alignments using Clustal Omega. Mol. Syst. Biol. 7, 539 (2011). 21988835 10.1038/msb.2011.75 PMC3261699

[b42] Felsenstein J. Confidence limits on phylogenies: an approach using the bootstrap. Evolution (N. Y). 39, 783–791 (1985). 10.1111/j.1558-5646.1985.tb00420.x 28561359

[b43] Larkin M. A. . Clustal W and Clustal X version 2.0. Bioinformatics 23, 2947–2948 (2007). 17846036 10.1093/bioinformatics/btm404

[b44] Letunic I. & Bork P. Interactive Tree Of Life (iTOL): an online tool for phylogenetic tree display and annotation. Bioinformatics 23, 127–128 (2007). 17050570 10.1093/bioinformatics/btl529

