# Supplementary material for: A new group of glycoside hydrolase family 13 α-amylases with an aberrant catalytic triad
Source: Sci Rep. 2017 Mar 13;7:44230. doi: 10.1038/srep44230 (PMC5347038; doi:10.1038/srep44230)
Supplement: Supplementary Dataset 1 [file srep44230-s1.doc]

Supplementary figures and tables

**A new group of glycoside hydrolase family 13 α-amylases with an aberrant catalytic triad**

**Fean D. Sarian ^1,2^**, **Štefan Janeček^3,4^, Tjaard** **Pijning^5^, Ihsanawati^1^**, **Zeily Nurachman^1^, Ocky K. radjasa^6^ , Lubbert Dijkhuizen^7^, Dessy Natalia^1^ and Marc J.E.C. van der Maarel^2*^**

**Supplementary Figure Legends**

**Figure S1.** **Topological alignment of the BmaN1 primary and secondary structure with GTA (PDB 4E20) and other putative amylases.** Alignment was prepared based on the super positioned models. Secondary structure: α-helix (green), TM-helix (golden), and β-sheet (blue); residues of the catalytic site (▲). Conserved secondary structures are named.

**Figure S2. Sequence alignment of α-amylases used in the present study.** The alignment spans the sequence segment from the beginning of the strand β2 (CSR-VI) to the end of the strand β8 (CSR-VII), i.e. a substantial part of the catalytic (β/α)_8_-barrel including domain B. The colour code for the sources of enzymes is explained in Table 2. Colour code for the individual residues: Trp - yellow; Phe, Tyr - blue; Asp, Glu - red; Arg, Lys - cyan; Val, Leu, Ile - green; His - brown; Cys - magenta; Gly, Pro - black. The catalytic machinery is signified by asterisks under the alignment blocks. The seven CSRs characteristic for the α-amylase family GH13 are emphasized by rectangles.

**Figure S3. SDS-PAGE(left) and activity staining after renaturation (right image) of BmaN1 purified from *B. megaterium* MS941.** M, molecular weight marker; lane 1, pMM1525 empty vector; lane2, BmaN1 protein. Activity staining was performed by soaking the starch-containing gel in 50 mM maleate buffer pH 6.0 and incubating at 55 ^o^C for 4 h. The clear bands after iodine solution treatment indicate amylolytic activity.

**Table 1. Primers used in amylase amplification.**

| Name | Sequence | Used |
| --- | --- | --- |
| BactF1 | AGAGTTTGATC(A/C)TGGCTCAG | 16s rDNA |
| UniB1 | GGTTAC(G/C)TTTGTTACGACTT | 16s rDNA |
| bmD_reg2F | GACGGATATTACGTTAAAGATATC | PCR of region VI-VII of *bmaN1* |
| bmD_reg6F | GGATTTACCGCTGTTTTATTAACTCC | PCR of region VI-VII of *bmaN1* |
| bmD_reg3R | ATTTCTCCTATTAATAGAAAAGAAGG | PCR of region VI-VII of *bmaN1* |
| bmD_reg7R | ATAAAAAACGATTGGTACACTCG | PCR of region VI-VII of *bmaN1* |
| NL3_SP8-invF1 | ACTGCTGCTACRTTTAGC | iPCR of *bmaN1* |
| NL3_SP8-invR1 | TATATKRCGCTCATGTGC | iPCR of *bmaN1* |
| pMM-NL3-F | GGATTCAGCAAGATCATAAAGATATACG | PCR of *bmaN1* |
| pET/MM-NL3-R | CGGCCGCTAGTGATGATGGTGATGGTGCGACGCGCTGTCCTTTTTACG | PCR of *bmaN1* |

Fig. S1

α2

Domain B

β1

β2

β3

α1

α3

**VI**

**I**

**V**


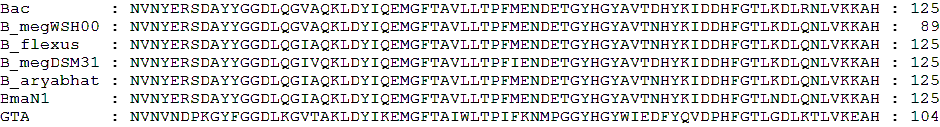

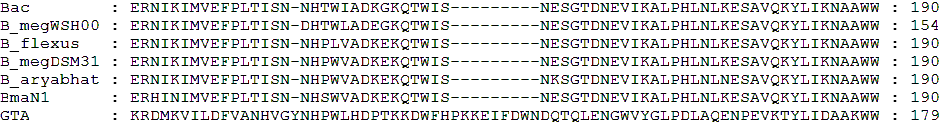

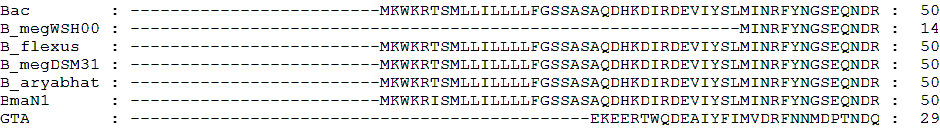

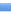

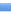

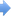

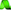

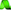

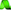

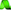

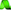

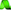

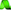

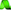

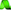

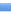

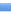

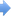

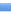

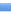

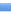

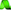

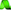

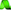

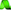

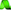

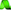

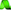

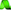

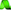

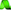

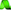

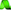

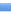

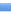

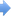

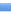

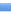

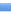

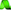

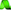

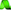

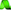

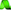

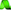

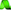

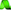

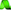

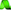

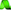

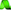

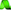

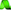

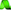

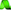

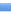

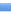

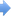

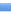

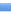

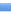

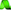

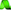

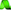

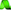

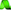

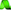

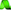

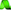

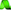

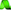

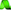

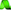

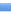

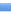

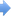

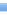

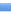

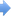

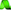

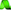

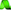

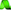

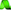

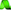

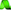

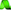

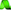

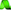

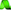

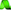

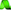

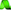

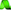

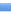

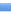

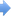

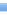

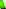

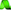


**II**

**III**

**IV**

β4

β5

β6

β7

β8

α4

α5

α6

α7

α8

**VII**

Fig S3.

**2 1 M 1 2**

(kDa)

**70**

**53**

**41**
